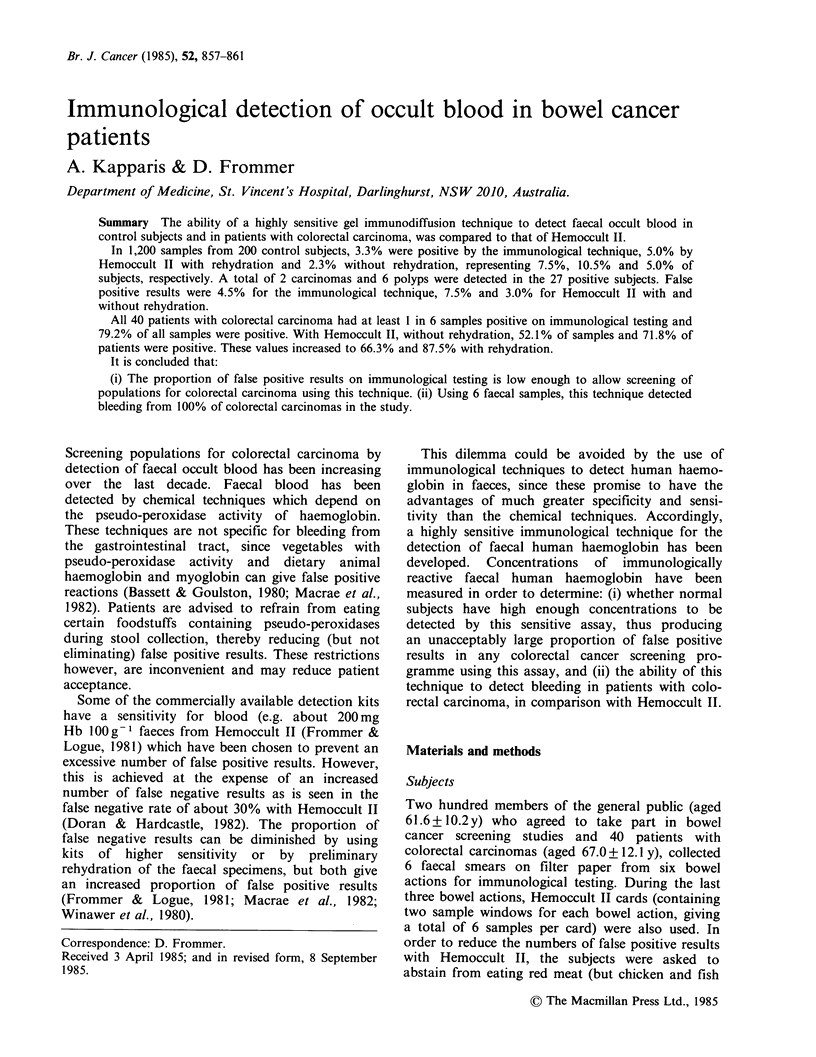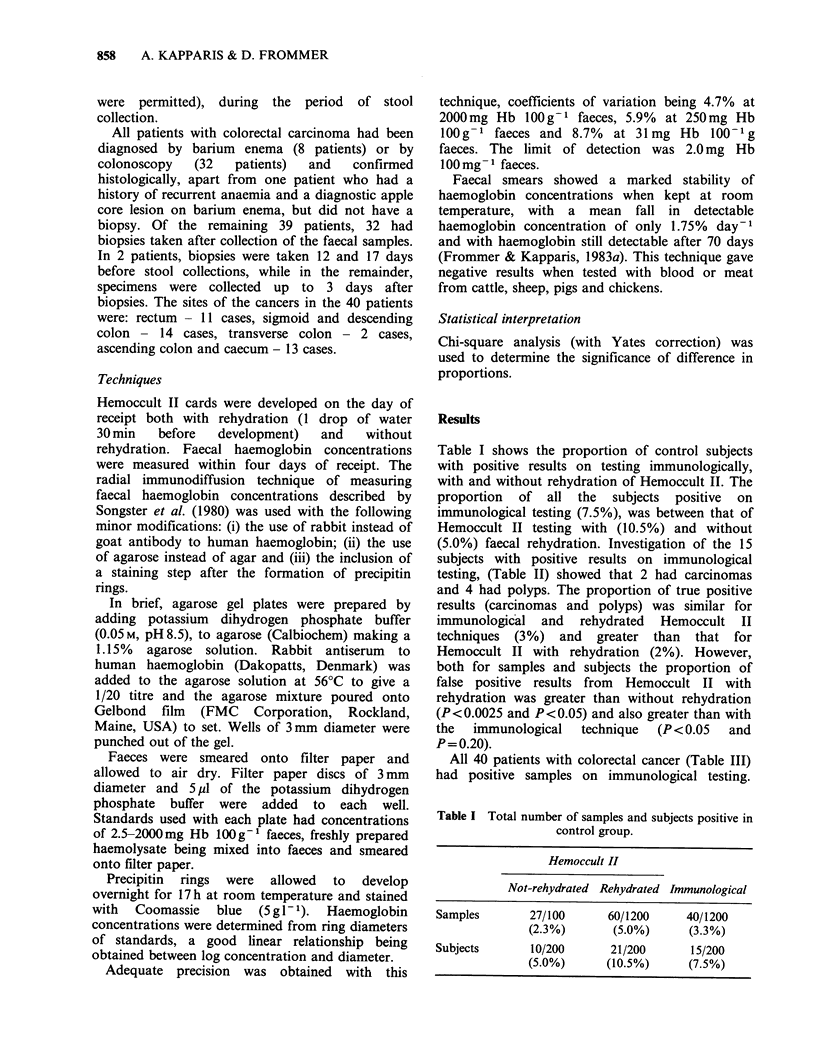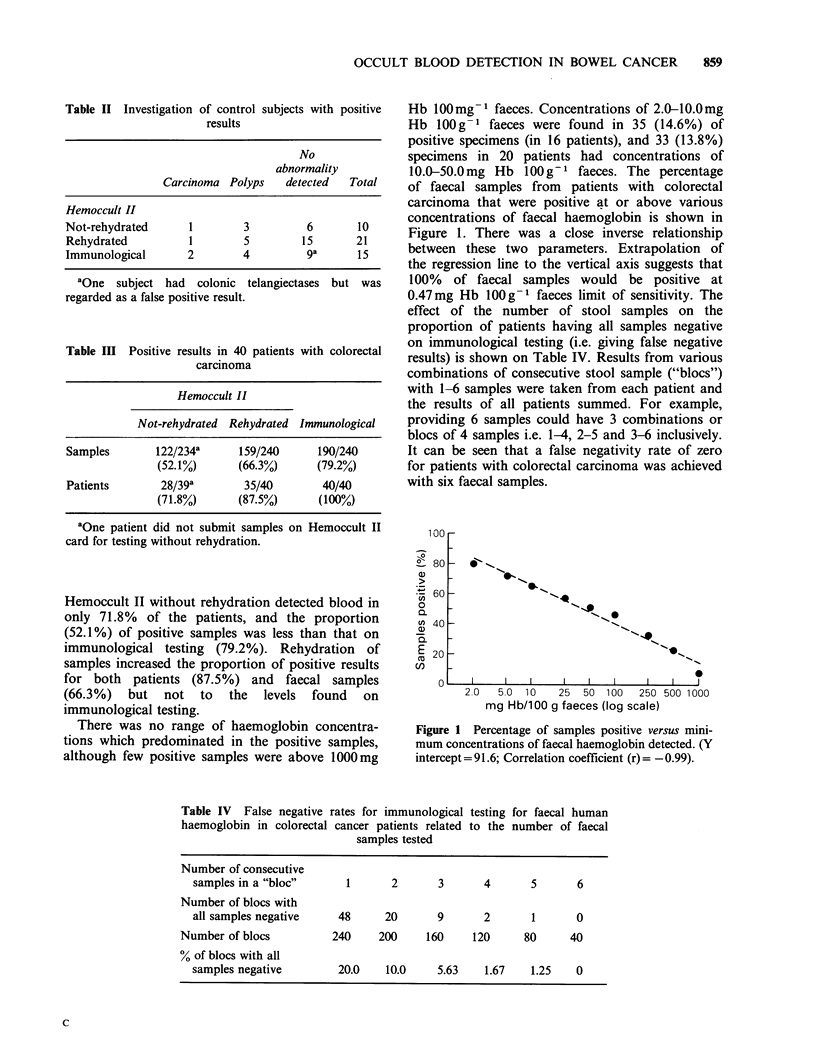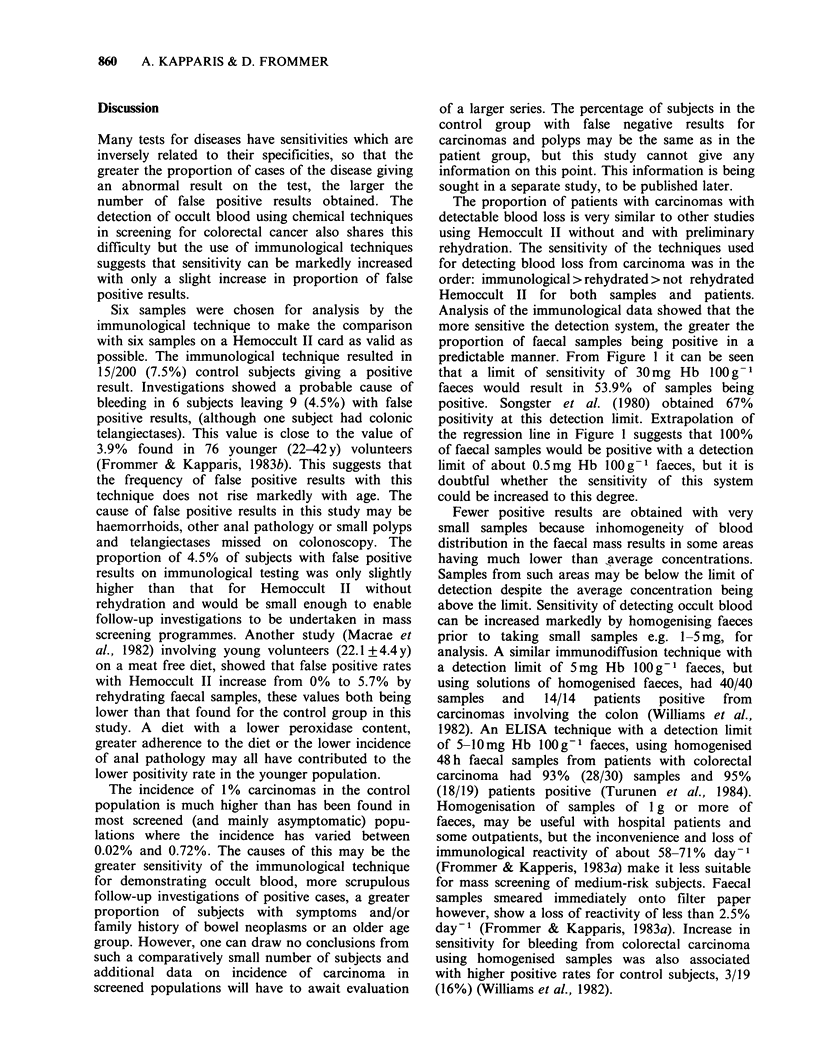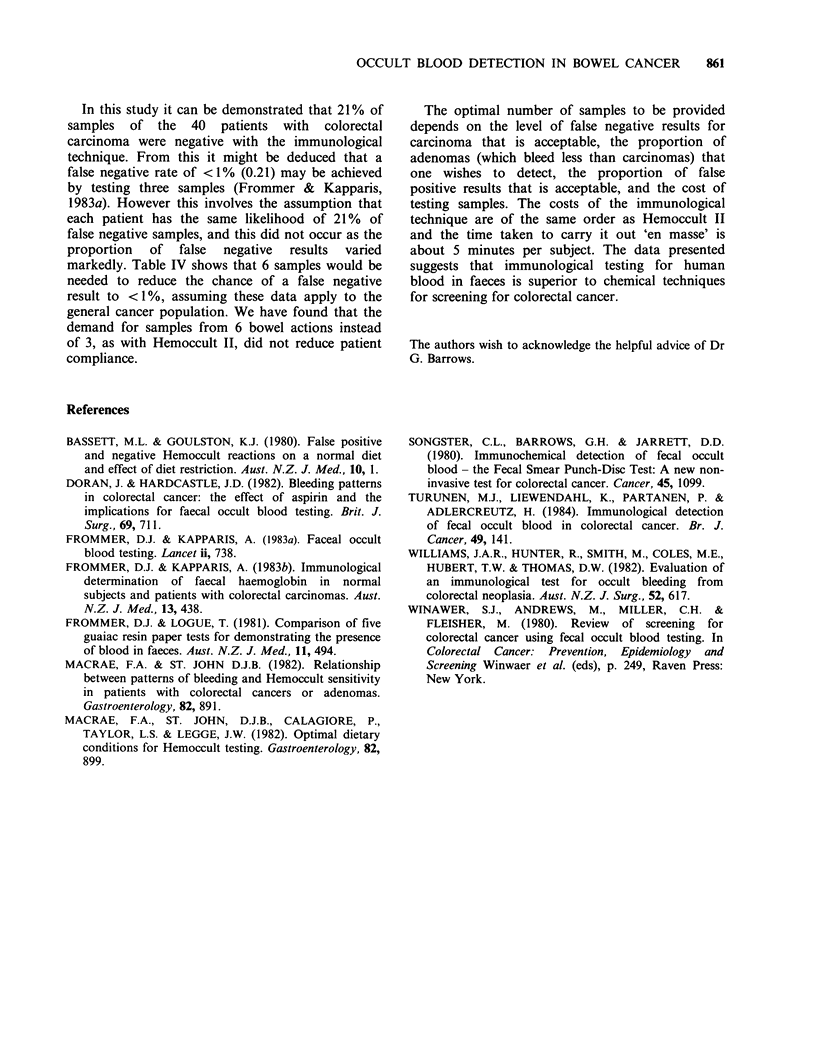# Immunological detection of occult blood in bowel cancer patients.

**DOI:** 10.1038/bjc.1985.269

**Published:** 1985-12

**Authors:** A. Kapparis, D. Frommer

## Abstract

The ability of a highly sensitive gel immunodiffusion technique to detect faecal occult blood in control subjects and in patients with colorectal carcinoma, was compared to that of Hemoccult II. In 1,200 samples from 200 control subjects, 3.3% were positive by the immunological technique, 5.0% by Hemoccult II with rehydration and 2.3% without rehydration, representing 7.5%, 10.5% and 5.0% of subjects, respectively. A total of 2 carcinomas and 6 polyps were detected in the 27 positive subjects. False positive results were 4.5% for the immunological technique, 7.5% and 3.0% for Hemoccult II with and without rehydration. All 40 patients with colorectal carcinoma had at least 1 in 6 samples positive on immunological testing and 79.2% of all samples were positive. With Hemoccult II, without rehydration, 52.1% of samples and 71.8% of patients were positive. These values increased to 66.3% and 87.5% with rehydration. It is concluded that: (i) The proportion of false positive results on immunological testing is low enough to allow screening of populations for colorectal carcinoma using this technique. (ii) Using 6 faecal samples, this technique detected bleeding from 100% of colorectal carcinomas in the study.


*Br. J. Cancer* (1985), **52**, 857–861

### Immunological detection of occult blood in bowel cancer patients

A. Kapparis & D. Frommer

*Department of Medicine, St. Vincent's Hospital, Darlinghurst, NSW 2010, Australia.*

**Summary** The ability of a highly sensitive gel immunodiffusion technique to detect faecal occult blood in control subjects and in patients with colorectal carcinoma, was compared to that of Hemoccult II.

In 1,200 samples from 200 control subjects, 3.3% were positive by the immunological technique, 5.0% by Hemoccult II with rehydration and 2.3% without rehydration, representing 7.5%, 10.5% and 5.0% of subjects, respectively. A total of 2 carcinomas and 6 polyps were detected in the 27 positive subjects. False positive results were 4.5% for the immunological technique, 7.5% and 3.0% for Hemoccult II with and without rehydration.

All 40 patients with colorectal carcinoma had at least 1 in 6 samples positive on immunological testing and 79.2% of all samples were positive. With Hemoccult II, without rehydration, 52.1% of samples and 71.8% of patients were positive. These values increased to 66.3% and 87.5% with rehydration.

It is concluded that:

(i) The proportion of false positive results on immunological testing is low enough to allow screening of populations for colorectal carcinoma using this technique. (ii) Using 6 faecal samples, this technique detected bleeding from 100% of colorectal carcinomas in the study.

Screening populations for colorectal carcinoma by detection of faecal occult blood has been increasing over the last decade. Faecal blood has been detected by chemical techniques which depend on the pseudo-peroxidase activity of haemoglobin. These techniques are not specific for bleeding from the gastrointestinal tract, since vegetables with pseudo-peroxidase activity and dietary animal haemoglobin and myoglobin can give false positive reactions (Bassett & Goulston, 1980; Macrae *et al.*, 1982). Patients are advised to refrain from eating certain foodstuffs containing pseudo-peroxidases during stool collection, thereby reducing (but not eliminating) false positive results. These restrictions however, are inconvenient and may reduce patient acceptance.

Some of the commercially available detection kits have a sensitivity for blood (e.g. about 200 mg Hb $100\,g^{-1}$ faeces from Hemoccult II (Frommer & Logue, 1981) which have been chosen to prevent an excessive number of false positive results. However, this is achieved at the expense of an increased number of false negative results as is seen in the false negative rate of about 30% with Hemoccult II (Doran & Hardcastle, 1982). The proportion of false negative results can be diminished by using kits of higher sensitivity or by preliminary rehydration of the faecal specimens, but both give an increased proportion of false positive results (Frommer & Logue, 1981; Macrae *et al.*, 1982; Winawer *et al.*, 1980).

Correspondence: D. Frommer.
Received 3 April 1985; and in revised form, 8 September 1985.

This dilemma could be avoided by the use of immunological techniques to detect human haemoglobin in faeces, since these promise to have the advantages of much greater specificity and sensitivity than the chemical techniques. Accordingly, a highly sensitive immunological technique for the detection of faecal human haemoglobin has been developed. Concentrations of immunologically reactive faecal human haemoglobin have been measured in order to determine: (i) whether normal subjects have high enough concentrations to be detected by this sensitive assay, thus producing an unacceptably large proportion of false positive results in any colorectal cancer screening programme using this assay, and (ii) the ability of this technique to detect bleeding in patients with colorectal carcinoma, in comparison with Hemoccult II.

### Materials and methods

### *Subjects*

Two hundred members of the general public (aged 61.6±10.2 y) who agreed to take part in bowel cancer screening studies and 40 patients with colorectal carcinomas (aged 67.0±12.1 y), collected 6 faecal smears on filter paper from six bowel actions for immunological testing. During the last three bowel actions, Hemoccult II cards (containing two sample windows for each bowel action, giving a total of 6 samples per card) were also used. In order to reduce the numbers of false positive results with Hemoccult II, the subjects were asked to abstain from eating red meat (but chicken and fish

© The Macmillan Press Ltd., 1985

were permitted), during the period of stool collection.

All patients with colorectal carcinoma had been diagnosed by barium enema (8 patients) or by colonoscopy (32 patients) and confirmed histologically, apart from one patient who had a history of recurrent anaemia and a diagnostic apple core lesion on barium enema, but did not have a biopsy. Of the remaining 39 patients, 32 had biopsies taken after collection of the faecal samples. In 2 patients, biopsies were taken 12 and 17 days before stool collections, while in the remainder, specimens were collected up to 3 days after biopsies. The sites of the cancers in the 40 patients were: rectum – 11 cases, sigmoid and descending colon – 14 cases, transverse colon – 2 cases, ascending colon and caecum – 13 cases.

### *Techniques*

Hemoccult II cards were developed on the day of receipt both with rehydration (1 drop of water 30 min before development) and without rehydration. Faecal haemoglobin concentrations were measured within four days of receipt. The radial immunodiffusion technique of measuring faecal haemoglobin concentrations described by Songster *et al.* (1980) was used with the following minor modifications: (i) the use of rabbit instead of goat antibody to human haemoglobin; (ii) the use of agarose instead of agar and (iii) the inclusion of a staining step after the formation of precipitin rings.

In brief, agarose gel plates were prepared by adding potassium dihydrogen phosphate buffer (0.05 M, pH 8.5), to agarose (Calbiochem) making a 1.15% agarose solution. Rabbit antiserum to human haemoglobin (Dakopatts, Denmark) was added to the agarose solution at 56°C to give a 1/20 titre and the agarose mixture poured onto Gelbond film (FMC Corporation, Rockland, Maine, USA) to set. Wells of 3 mm diameter were punched out of the gel.

Faeces were smeared onto filter paper and allowed to air dry. Filter paper discs of 3 mm diameter and 5 $\mu l$ of the potassium dihydrogen phosphate buffer were added to each well. Standards used with each plate had concentrations of 2.5–2000 mg Hb 100 $g^{-1}$ faeces, freshly prepared haemolysate being mixed into faeces and smeared onto filter paper.

Precipitin rings were allowed to develop overnight for 17 h at room temperature and stained with Coomassie blue (5 g $l^{-1}$). Haemoglobin concentrations were determined from ring diameters of standards, a good linear relationship being obtained between log concentration and diameter.

Adequate precision was obtained with this technique, coefficients of variation being 4.7% at 2000 mg Hb 100 $g^{-1}$ faeces, 5.9% at 250 mg Hb 100 $g^{-1}$ faeces and 8.7% at 31 mg Hb $100^{-1}$ g faeces. The limit of detection was 2.0 mg Hb 100 $mg^{-1}$ faeces.

Faecal smears showed a marked stability of haemoglobin concentrations when kept at room temperature, with a mean fall in detectable haemoglobin concentration of only 1.75% $day^{-1}$ and with haemoglobin still detectable after 70 days (Frommer & Kapparis, 1983*a*). This technique gave negative results when tested with blood or meat from cattle, sheep, pigs and chickens.

### *Statistical interpretation*

Chi-square analysis (with Yates correction) was used to determine the significance of difference in proportions.

### Results

Table I shows the proportion of control subjects with positive results on testing immunologically, with and without rehydration of Hemoccult II. The proportion of all the subjects positive on immunological testing (7.5%), was between that of Hemoccult II testing with (10.5%) and without (5.0%) faecal rehydration. Investigation of the 15 subjects with positive results on immunological testing, (Table II) showed that 2 had carcinomas and 4 had polyps. The proportion of true positive results (carcinomas and polyps) was similar for immunological and rehydrated Hemoccult II techniques (3%) and greater than that for Hemoccult II with rehydration (2%). However, both for samples and subjects the proportion of false positive results from Hemoccult II with rehydration was greater than without rehydration ($P<0.0025$ and $P<0.05$) and also greater than with the immunological technique ($P<0.05$ and $P=0.20$).

All 40 patients with colorectal cancer (Table III) had positive samples on immunological testing.

**Table I** Total number of samples and subjects positive in control group.

| | *Hemoccult II* | | |
|---|---|---|---|
| | *Not-rehydrated* | *Rehydrated* | *Immunological* |
| Samples | 27/100 (2.3%) | 60/1200 (5.0%) | 40/1200 (3.3%) |
| Subjects | 10/200 (5.0%) | 21/200 (10.5%) | 15/200 (7.5%) |

**Table II** Investigation of control subjects with positive results

| | Carcinoma | Polyps | No abnormality detected | Total |
|---|---|---|---|---|
| *Hemoccult II* | | | | |
| Not-rehydrated | 1 | 3 | 6 | 10 |
| Rehydrated | 1 | 5 | 15 | 21 |
| Immunological | 2 | 4 | 9[a] | 15 |

[a]One subject had colonic telangiectases but was regarded as a false positive result.

**Table III** Positive results in 40 patients with colorectal carcinoma

| | *Hemoccult II* | | |
|---|---|---|---|
| | *Not-rehydrated* | *Rehydrated* | *Immunological* |
| Samples | 122/234[a] (52.1%) | 159/240 (66.3%) | 190/240 (79.2%) |
| Patients | 28/39[a] (71.8%) | 35/40 (87.5%) | 40/40 (100%) |

[a]One patient did not submit samples on Hemoccult II card for testing without rehydration.

Hemoccult II without rehydration detected blood in only 71.8% of the patients, and the proportion (52.1%) of positive samples was less than that on immunological testing (79.2%). Rehydration of samples increased the proportion of positive results for both patients (87.5%) and faecal samples (66.3%) but not to the levels found on immunological testing.

There was no range of haemoglobin concentrations which predominated in the positive samples, although few positive samples were above 1000 mg Hb 100 mg$^{-1}$ faeces. Concentrations of 2.0–10.0 mg Hb 100 g$^{-1}$ faeces were found in 35 (14.6%) of positive specimens (in 16 patients), and 33 (13.8%) specimens in 20 patients had concentrations of 10.0–50.0 mg Hb 100 g$^{-1}$ faeces. The percentage of faecal samples from patients with colorectal carcinoma that were positive at or above various concentrations of faecal haemoglobin is shown in Figure 1. There was a close inverse relationship between these two parameters. Extrapolation of the regression line to the vertical axis suggests that 100% of faecal samples would be positive at 0.47 mg Hb 100 g$^{-1}$ faeces limit of sensitivity. The effect of the number of stool samples on the proportion of patients having all samples negative on immunological testing (i.e. giving false negative results) is shown on Table IV. Results from various combinations of consecutive stool sample ("blocs") with 1–6 samples were taken from each patient and the results of all patients summed. For example, providing 6 samples could have 3 combinations or blocs of 4 samples i.e. 1–4, 2–5 and 3–6 inclusively. It can be seen that a false negativity rate of zero for patients with colorectal carcinoma was achieved with six faecal samples.

**Figure 1** Percentage of samples positive *versus* minimum concentrations of faecal haemoglobin detected. (Y intercept = 91.6; Correlation coefficient (r) = −0.99).

**Table IV** False negative rates for immunological testing for faecal human haemoglobin in colorectal cancer patients related to the number of faecal samples tested

| | | | | | | |
|---|---|---|---|---|---|---|
| Number of consecutive samples in a "bloc" | 1 | 2 | 3 | 4 | 5 | 6 |
| Number of blocs with all samples negative | 48 | 20 | 9 | 2 | 1 | 0 |
| Number of blocs | 240 | 200 | 160 | 120 | 80 | 40 |
| % of blocs with all samples negative | 20.0 | 10.0 | 5.63 | 1.67 | 1.25 | 0 |

### Discussion

Many tests for diseases have sensitivities which are inversely related to their specificities, so that the greater the proportion of cases of the disease giving an abnormal result on the test, the larger the number of false positive results obtained. The detection of occult blood using chemical techniques in screening for colorectal cancer also shares this difficulty but the use of immunological techniques suggests that sensitivity can be markedly increased with only a slight increase in proportion of false positive results.

Six samples were chosen for analysis by the immunological technique to make the comparison with six samples on a Hemoccult II card as valid as possible. The immunological technique resulted in 15/200 (7.5%) control subjects giving a positive result. Investigations showed a probable cause of bleeding in 6 subjects leaving 9 (4.5%) with false positive results, (although one subject had colonic telangiectases). This value is close to the value of 3.9% found in 76 younger (22–42 y) volunteers (Frommer & Kapparis, 1983*b*). This suggests that the frequency of false positive results with this technique does not rise markedly with age. The cause of false positive results in this study may be haemorrhoids, other anal pathology or small polyps and telangiectases missed on colonoscopy. The proportion of 4.5% of subjects with false positive results on immunological testing was only slightly higher than that for Hemoccult II without rehydration and would be small enough to enable follow-up investigations to be undertaken in mass screening programmes. Another study (Macrae *et al.*, 1982) involving young volunteers ($22.1 \pm 4.4$ y) on a meat free diet, showed that false positive rates with Hemoccult II increase from 0% to 5.7% by rehydrating faecal samples, these values both being lower than that found for the control group in this study. A diet with a lower peroxidase content, greater adherence to the diet or the lower incidence of anal pathology may all have contributed to the lower positivity rate in the younger population.

The incidence of 1% carcinomas in the control population is much higher than has been found in most screened (and mainly asymptomatic) populations where the incidence has varied between 0.02% and 0.72%. The causes of this may be the greater sensitivity of the immunological technique for demonstrating occult blood, more scrupulous follow-up investigations of positive cases, a greater proportion of subjects with symptoms and/or family history of bowel neoplasms or an older age group. However, one can draw no conclusions from such a comparatively small number of subjects and additional data on incidence of carcinoma in screened populations will have to await evaluation of a larger series. The percentage of subjects in the control group with false negative results for carcinomas and polyps may be the same as in the patient group, but this study cannot give any information on this point. This information is being sought in a separate study, to be published later.

The proportion of patients with carcinomas with detectable blood loss is very similar to other studies using Hemoccult II without and with preliminary rehydration. The sensitivity of the techniques used for detecting blood loss from carcinoma was in the order: immunological > rehydrated > not rehydrated Hemoccult II for both samples and patients. Analysis of the immunological data showed that the more sensitive the detection system, the greater the proportion of faecal samples being positive in a predictable manner. From Figure 1 it can be seen that a limit of sensitivity of 30 mg Hb 100 $g^{-1}$ faeces would result in 53.9% of samples being positive. Songster *et al.* (1980) obtained 67% positivity at this detection limit. Extrapolation of the regression line in Figure 1 suggests that 100% of faecal samples would be positive with a detection limit of about 0.5 mg Hb 100 $g^{-1}$ faeces, but it is doubtful whether the sensitivity of this system could be increased to this degree.

Fewer positive results are obtained with very small samples because inhomogeneity of blood distribution in the faecal mass results in some areas having much lower than average concentrations. Samples from such areas may be below the limit of detection despite the average concentration being above the limit. Sensitivity of detecting occult blood can be increased markedly by homogenising faeces prior to taking small samples e.g. 1–5 mg, for analysis. A similar immunodiffusion technique with a detection limit of 5 mg Hb 100 $g^{-1}$ faeces, but using solutions of homogenised faeces, had 40/40 samples and 14/14 patients positive from carcinomas involving the colon (Williams *et al.*, 1982). An ELISA technique with a detection limit of 5–10 mg Hb 100 $g^{-1}$ faeces, using homogenised 48 h faecal samples from patients with colorectal carcinoma had 93% (28/30) samples and 95% (18/19) patients positive (Turunen *et al.*, 1984). Homogenisation of samples of 1 g or more of faeces, may be useful with hospital patients and some outpatients, but the inconvenience and loss of immunological reactivity of about 58–71% $day^{-1}$ (Frommer & Kapperis, 1983*a*) make it less suitable for mass screening of medium-risk subjects. Faecal samples smeared immediately onto filter paper however, show a loss of reactivity of less than 2.5% $day^{-1}$ (Frommer & Kapparis, 1983*a*). Increase in sensitivity for bleeding from colorectal carcinoma using homogenised samples was also associated with higher positive rates for control subjects, 3/19 (16%) (Williams *et al.*, 1982).

In this study it can be demonstrated that 21% of samples of the 40 patients with colorectal carcinoma were negative with the immunological technique. From this it might be deduced that a false negative rate of <1% (0.21) may be achieved by testing three samples (Frommer & Kapparis, 1983*a*). However this involves the assumption that each patient has the same likelihood of 21% of false negative samples, and this did not occur as the proportion of false negative results varied markedly. Table IV shows that 6 samples would be needed to reduce the chance of a false negative result to <1%, assuming these data apply to the general cancer population. We have found that the demand for samples from 6 bowel actions instead of 3, as with Hemoccult II, did not reduce patient compliance.

The optimal number of samples to be provided depends on the level of false negative results for carcinoma that is acceptable, the proportion of adenomas (which bleed less than carcinomas) that one wishes to detect, the proportion of false positive results that is acceptable, and the cost of testing samples. The costs of the immunological technique are of the same order as Hemoccult II and the time taken to carry it out 'en masse' is about 5 minutes per subject. The data presented suggests that immunological testing for human blood in faeces is superior to chemical techniques for screening for colorectal cancer.

The authors wish to acknowledge the helpful advice of Dr G. Barrows.

### References

BASSETT, M.L. & GOULSTON, K.J. (1980). False positive and negative Hemoccult reactions on a normal diet and effect of diet restriction. *Aust. N.Z. J. Med.*, **10**, 1.

DORAN, J. & HARDCASTLE, J.D. (1982). Bleeding patterns in colorectal cancer: the effect of aspirin and the implications for faecal occult blood testing. *Brit. J. Surg.*, **69**, 711.

FROMMER, D.J. & KAPPARIS, A. (1983*a*). Faceal occult blood testing. *Lancet* **ii**, 738.

FROMMER, D.J. & KAPPARIS, A. (1983*b*). Immunological determination of faecal haemoglobin in normal subjects and patients with colorectal carcinomas. *Aust. N.Z. J. Med.*, **13**, 438.

FROMMER, D.J. & LOGUE, T. (1981). Comparison of five guaiac resin paper tests for demonstrating the presence of blood in faeces. *Aust. N.Z. J. Med.*, **11**, 494.

MACRAE, F.A. & ST. JOHN D.J.B. (1982). Relationship between patterns of bleeding and Hemoccult sensitivity in patients with colorectal cancers or adenomas. *Gastroenterology*, **82**, 891.

MACRAE, F.A., ST. JOHN, D.J.B., CALAGIORE, P., TAYLOR, L.S. & LEGGE, J.W. (1982). Optimal dietary conditions for Hemoccult testing. *Gastroenterology*, **82**, 899.

SONGSTER, C.L., BARROWS, G.H. & JARRETT, D.D. (1980). Immunochemical detection of fecal occult blood – the Fecal Smear Punch-Disc Test: A new non-invasive test for colorectal cancer. *Cancer*, **45**, 1099.

TURUNEN, M.J., LIEWENDAHL, K., PARTANEN, P. & ADLERCREUTZ, H. (1984). Immunological detection of fecal occult blood in colorectal cancer. *Br. J. Cancer*, **49**, 141.

WILLIAMS, J.A.R., HUNTER, R., SMITH, M., COLES, M.E., HUBERT, T.W. & THOMAS, D.W. (1982). Evaluation of an immunological test for occult bleeding from colorectal neoplasia. *Aust. N.Z. J. Surg.*, **52**, 617.

WINAWER, S.J., ANDREWS, M., MILLER, C.H. & FLEISHER, M. (1980). Review of screening for colorectal cancer using fecal occult blood testing. In *Colorectal Cancer: Prevention, Epidemiology and Screening* Winwaer *et al.* (eds), p. 249, Raven Press: New York.